# Right heart exercise-training-adaptation and remodelling in endurance athletes

**DOI:** 10.1038/s41598-021-02028-1

**Published:** 2021-11-18

**Authors:** Valeria Conti, Filippo Migliorini, Marco Pilone, María I. Barriopedro, Juan José Ramos-Álvarez, Francisco Javer Calderon Montero, Nicola Maffulli

**Affiliations:** 1 Department of Medicine, Surgery and Dentistry, University of Salerno, Baronissi, Italy; 2 Department of Orthopaedic, Trauma, and Recontructive Surgery, RWTH Aachen University Clinic, Pauwelsstraße 30, 52074 Aachen, Germany; 3 Faculty of Physical Activity and Sport Sciences, Universidad Politécnica de Madrid, Madrid, Spain; 4 Escuela de Medicina Deportiva. Departamento de Radiología, Rehabilitación y Fisioterapia, Universidad Complutense de Madrid, Madrid, Spain; 5 Centre for Sports and Exercise Medicine, Barts and The London School of Medicine and Dentistry, Mile End Hospital, Queen Mary University of London, 275 Bancroft Road, London, E1 4DG England, UK; 6 Department of Musculoskeletal Disorders, School of Medicine, Surgery and Dentistry, University of Salerno, Salerno, Italy

**Keywords:** Cardiology, Medical research

## Abstract

Long-term sports training leads to myocardial adaptations, with remodelling of the heart chambers. However, while myocardial adaptations of the left heart are well described, remodelling of the right heart and its impact on the development of arrhythmias is still debated. To conduct a systematic review on right ventricle (RV) and right atrium (RA) structural and functional changes in athletes who participate in long-term endurance training. Systematic review. A systematic literature search was conducted. All the articles reporting right heart echocardiographic (ECHO) and cardiac magnetic resonance (CMR) parameters evaluated in endurance athletes and sedentary subjects were considered eligible. A multivariate analysis was conducted to investigate whether age, sex, body surface area (BSA), intensity of training are associated with RV ECHO, CMR parameters and RA ECHO parameters. A positive association between age and right atrium area (RAA) (*P* = 0.01) was found. This is a negative association to RV E/A (*P* = 0.004), and RV end diastolic diameter (RVED) longitudinal (*P* = 0.01). A positive association between BSA and RVED middle (*P* = 0.001), as well between BSA and RAA (*P* = 0.05) was found, along with a negative association with RV E/A (*P* = 0.002). A positive association between intensity of training and RV end systolic area (RVESA) (*P* = 0.03), RV end diastolic volume indexed (RVEDVI) (*P* = 0.01), RV end systolic volume indexed (RVESVI) (*P* = 0.01) was found, along with a negative association with ejection fraction (EF %) (*P* = 0.01). Endurance athletes demonstrated an association between RV remodelling and age, BSA and intensity of training.

## Introduction

“Athlete’s heart” refers to remodelling of the whole cardiac muscles induced by the high workload of sports training^1–10^. Heart remodelling is different following endurance training and resistance training^11^.

In endurance training, the left ventricle (LV) undergoes eccentric hypertrophy, while resistance training promotes concentric hypertrophy^12^. A clear classification is not possible as the overall size of cardiomyocytes increases under both remodelling patterns. In general, the thickness of the LV wall prevails in resistance training as a consequence of chronic volume overload; LV dilatation is a prominent characteristic in endurance-trained heart, mainly determined by chronic systolic pressure overload^13^.

However, exercise-induced changes of the right ventricle (RV) are still debated^14^. Also, there seems to be an association between RV hypertrophy and Arrhythmogenic Right Ventricular Cardiomyopathy (ARVC)^15–17^, one of the major causes of cardiac death in young athletes^18,19^. Therefore, it would be interesting to define exercise-induced RV adaptation, and its impact on the onset of arrhythmias^16^. Endurance training causes greater morphological and functional RV changes than any other type of exercise, but the role of variables such as age, sex and BSA has not been established^20^. Endurance training increases the hemodynamic needs with a consequent chronic pressure elevation in the cardiac chambers^21^. As a result, long-term exercise leads to permanent RV structural and functional modifications, with large interindividual variability^22^. It is important to distinguish between acute and chronic adaptations, and to consider exercise loading and intensity^23^, which induce different functional and clinical consequences^22,23^. Immediately after an intense exercise, the serum level of troponin and B-type natriuretic peptide increases^17,24^. A process of micro injuring and healing occurs, mirroring what take place in skeletal muscles, which, eventually, leads to myocardial adaptation^22^.

This study investigates the structural and functional changes of the right ventricle (RV) and right atrium (RA) in athletes who participate in long-term endurance training, and defines whether the RV ECHO and CMR reference values for the general population are appropriate for endurance athletes.

## Methods

### Search strategy

The present systematic review was conducted according to the Preferred Reporting Items for Systematic Reviews and Meta-analyses (PRISMA) recomendations^25^. We followed the PICO protocol for the preliminary search:

- P (Problem): right heart in endurance athletes;
- I (Intervention): long-term endurance sport training;
- C (Comparator): right heart in sedentary individuals;
- O (Outcomes): morphological and functional ECHO and CMR parameters.

### Literature search and selection

Two authors (MP; VC) independently performed the literature search in May 2021. The following databases were used for the search: Medline, Scopus and Cochrane. To conduct a comprehensive systematic literature search, we used both controlled vocabulary and free text terms. For our research, the following MESH terms were used: *right ventricle, right atrium, right heart, heart chambers, endurance, athletes, echocardiography, cardiac magnetic resonance, sport, players, remodelling, adaptation, training, work out*. Only studies published from 2000 up to May 2021 were accessed. Resulting titles and abstracts of interests were screened by the two authors independently. The full text of the articles of interest were examined. Disagreements about the eligibility of a study were resolved were solved by a third author (FM).

### Eligibility criteria

According to the authors’ language capabilities, articles in English, Italian, French, German, Portuguese and Spanish were considered. Studies with level of evidence I to III, according to the Oxford Centre for Evidence-Based Medicine (OCEBM), were considered^26^. Data from national registries were excluded, as well as reviews, letters, expert opinions, case reports, editorials, animal, computational and cadaveric studies. As the aim was to investigate the remodelling of the right heart in endurance athletes, only study which investigated athletes who regularly played sports listed on the Mitchel Category class C^27^ were examined. Only studies reporting right heart ECHO and/or CMR parameters of endurance athletes and a sedentary group as controls were eligible. To evaluate chronic adaptations, only CMR or an ECHO evaluation performed at rest were considered. The exclusions criteria were represented by all the variables which could alter the physiological parameters: the presence of diseases, a family history of pulmonary or cardiac diseases, chronic use of drugs.

### Data extraction

Data were extracted by two different authors (MP; VC). Data from the following end-points were collected:

- generalities displayed author and publication year and general characteristics of our population: number of athletes, number of sedentary individuals, sex, age, body mass index (BMI), body surface area (BSA), heart rate (HR), training hours and years of training.
- ECHO data to assess the morphological and functional parameters of the right heart: basal, middle, longitudinal right ventricle end diastolic diameter (RVED) and their values BSA indexed, right ventricle end diastolic area (RVEDA), end systolic area (RVESA) and their values BSA indexed, right ventricle wall thickness (RVWT), right ventricle E/A (RV E/A), fractional area change (FAC %), ejection fraction (EF %) tricuspid annular plane excursion (TAPSE), right ventricle outflow tract (RVOT1,RVOT2,RVOT3) and their values BSA indexed, right atrial area (RAA), right atrial volume (RAV) and their value BSA indexed.
- CMR data to assess the morphological and functional parameters of the right heart: right ventricle end diastolic volume (RVEDV), right ventricle end systolic volume (RVESV), right ventricle stroke volume (RVSV), mass and their value BSA indexed and ejection fraction (EF %).

### Outcomes of interest

The primary outcome of interest was to investigate the association between right heart remodelling and age, sex, BSA and intensity of training. The second outcome of interest was to compare the mean values of endurance athletes morphological and functional parameters. To underline the different adaptation of RV in male and female among CMR studies, five studies were selected, in which parameters referred to male athletes, male sedentary, female athletes, female sedentary were reported separately. In these studies, the difference of the means between male athletes-male sedentary subjects and female athletes-female sedentary subjects were compared.

### Methodological quality assessment

The same two reviewers who extracted data (MP; VC) assessed the risk of bias of the included studies using the Newcastle Ottawa Scale (NOS)^28^. The NOS evaluates three parameters, namely selection, comparability and exposure. The parameter selection domain includes 4 items useful to check definition and representativeness of cases and controls; the comparability domain includes 1 item to compare cases and controls based on the study design or analysis; the exposure domain includes 3 items to evaluate methods adopted in the study to ascertain exposure for cases and controls^28^. The maximum score for each study is nine points, and a study with lesser than five points is considered at high risk of bias. The NOS has been described as a reliable tool to assess the quality of case control studies and cohort studies^29^.

### Statistical analysis

For the multivariate analyses, the STATA/MP 16.1 (StataCorp, College Station, TX) was used, with a multiple linear model regression diagnostic. For pairwise correlation, the Pearson Product-Moment Correlation Coefficient (r) was used. The final effect was evaluated according to the Cauchy–Schwarz inequality: + 1 (positive linear correlation) and − 1 (negative linear correlation). Values of 0.1 <| *r* |< 0.3, 0.3 <| *r* |< 0.5, and | *r* |> 0.5 were considered to have small, medium, and strong correlation, respectively. The test of overall significance was performed through the χ2 test, with values of P < 0.05 considered statistically significant. For continuous variable, mean and standard deviation was evaluated. The T test was performed, with P < 0.05 considered statistically significant.

## Results

### Search results

**Figure 1:**
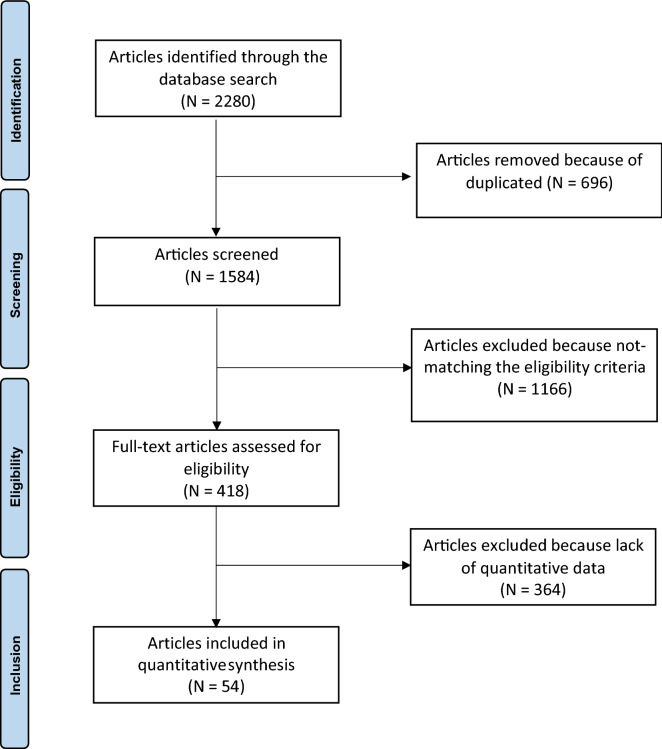
Flow-chart of literature search.

The search resulted in 2280 articles. After removing duplicates (N = 696), a total of 1584 articles were screened. A further 1166 articles were excluded because of: the type of the study (N = 508), the absence of sedentary controls (N = 193), sports not listed on the Mitchel Category class C (N = 131), language limitations (N = 7), evaluation after an acute stress (N = 118), presence of diseases (N = 209). Another 364 studies were excluded because they did not match the topic of interest or did not report quantitative data on outcome of interest. Finally, 54 articles were included in the analysis: 32 articles reporting ECHO parameters, 20 reporting CMR parameters, 2 reporting both. The flowchart of the literature search is shown in Fig. 1.

### Methodological quality assessment

The NOS scale evidenced an overall high quality of the included studies. Studies eligibility criteria and sports category were well described, as were the selection criteria of control groups. The intensity of training was seldom reported. Demographic characteristics were often exhaustively described. The procedure to assess outcomes was overall clearly described. The description of ECHO and CMR results was also comprehensive. A NOS scale score > 8 was achieved by all the included studies.

### Population demographic

In ECHO studies, data from 2102 athletes and 1311 sedentary practice were collected. The mean age of athletes and sedentary was 26.6 ± 8.6 and 26.6 ± 7.6, respectively. The BSA was 1.86 ± 0.2 and 1.82 ± 0.15, respectively. The BMI of athletes was 24.8 ± 2.01 and BMI of sedentary was 22.89 ± 1.32. HR was 57.3 ± 7.06 and 69.6 ± 5.26, respectively. In CMR studies, data from 1257 athletes and 909 sedentary subjects were collected. The mean age of athletes and sedentary was 31.5 ± 9.8 and 31.9 ± 8.8, respectively. The BSA was 1.89 ± 0.19 and 1.87 ± 0.15, respectively. The BMI of athletes was 22.4 ± 0.5, and that of sedentary participants was 24.4 ± 1.1. HR was 57.3 ± 7.06 and 63.4 ± 6.9, respectively. Population demographics is shown in Table 1 and in Table 2.

**Table 1:** Population demographics in ECHO studies.

Author name	N Athletes	N Sedentary	M% Athletes	Age Athletes	Age sedentary	BMI athletes (h/m^2^)	BMI Sedentary (h/m^2^)	BSA Athletes (m^2^)	BSA Sedentary (m^2^)	HR Athletes	HR Sedentary	Intensity of training (h/w)	Years of training
Baggish 2010^38^	20	20	100%	25 ± 3	21 ± 2	NA	NA	2.3 ± 0.1	2.0 ± 0.1	56 ± 7	72 ± 8	22 ± 6	9 ± 4
Baggish 2010^38^ ◄	20		100%	20 ± 2		NA	NA	2.1 ± 0.2		60 ± 5		11 ± 4	5 ± 2
Bauce 2008^31^	40	40	70%	26 ± 5	28 ± 12	NA	NA	1.84 ± 0.2	1.80 ± 0.1	NA	NA	7 ± 1.4	5.2
Bernheim 2013^1^	39	23	82%	36 (31–43)	36 (31–43)	NA	NA	1.3(1.8–2.0)	1.98 (1.75–2.05)	56(50–64)	73 (65–81)	13.5 ± 3.5	NA
Bjerring 2018°^39^	76	25	63%	12.1 ± 0.2	12.1 ± 0.3	17.5 ± 1.5	19.9 ± 4.0	1.32 ± 0.12	1.43 ± 0.21	72 ± 12	80 ± 20	7 ± 2.3	5.4 ± 1.2
D'Andrea2013^3^	395	230	61%	28.8 ± 10.1	27.5 ± 11.3	NA	NA	1.84 ± 0.5	1.83 ± 0.6	52.1 ± 4.4	70.5 ± 10.6	15–20	> 4
D'Ascenzi 2012^40^	100	78	76%	25.91 ± 4.61	25.20 ± 3.92	NA	NA	2.05 ± 0.23	1.82 ± 0.18	60.69 ± 9.63	72.16 ± 13.1	> 15	5
D'Ascenzi 2016^41^°	57	37	100%	10.8 ± 0.2	10.2 ± 0.2	NA	NA	1.34 ± 0.20	1.33 ± 0.20	78 ± 11	67 ± 9	> 10	NA
De Luca 2013^42^	25	20	100%	20 ± 3	18.3 ± 3.6	NA	NA	NA	NA	70.3 ± 9.04	77.9 ± 8.52	> 10	10 ± 3
Doronina 2018	15	15	0%	24 ± 4	23 ± 2	21.4 ± 1.2	20.5 ± 2.8	1.8 ± 0.1	1.6 ± 0.1	69 ± 14	82 ± 7	24 ± 8	12.1 ± 4.6
Esposito 2014^43^	40	43	100%	28 ± 9.5	29.0 ± 5.8	24.2 ± 2.5	23.2 ± 3.0	NA	NA	55.3 ± 8.0	68.5 ± 10.6	> 30	> 4
Eun 2016^44^	29	29	100%	27 ± 4.1	26 ± 6.5	NA	NA	1.97 ± 0.1	1.87 ± 0.2	54.2 ± 10.1	73.3 ± 10.7	ELITE	NA
Hedman 2014^45^	46	48	0%	21 ± 2	21 ± 2	NA	NA	1.69 ± 0.10	1.63 ± 0.09	54 ± 8	71 ± 10	13 ± 2	6 ± 2
King2013^46^	24	17	100%	24.3 ± 4.2	27.06 ± 3.5	NA	NA	2.0 ± 0.2	1.93 ± 0.2	61.29 ± 9.31	71.71 ± 9.31	> 10	NA
King2013^46^◄	18		100%	22.38 ± 3.7		NA	NA	2.08 ± 0.13		61.72 ± 8.59	> 8	NA	
Koc 2007^2^	60	60	90%	20.7 ± 2.5	21.3 ± 2.6	NA	NA	2.0 ± 0.2	1.9 ± 0.2	65.4 ± 8.7	77.0 ± 7.8	15 ± 5	> 5
Kooreman2019^47^	37	31	0%	19 ± 1	19 ± 1	22.1 ± 2.3	23.5 ± 3.5	1.8 ± 0.2	1.7 ± 0.2	NA	NA	ELITE	NA
La Gerche 2015^16^	10	7	100%	35 ± 6	34 ± 16	22.9 ± 1.5	23.2 ± 1.7	NA	NA	52 ± 8	62 ± 12	11 (6- 1 5)	9 (6–17)
Lakatos 2018^48^	30	20	100%	18.9 ± 4.0	19.9 ± 3.8	NA	NA	2.2 ± 0.2	1.9 ± 0.2	67.2 ± 11.5	68.4 ± 9.1	17 ± 6	10 ± 5
Lakatos2018^48^ ♀	30	20	0%	19.0 ± 3.7	19.5 ± 2.3	NA	NA	1.8 ± 0.1	1.6 ± 0.1	71.1 ± 12.1	84.2 ± 19.2	17 ± 6	10 ± 5
Leischik 2016^49^	33	37	0%	34.3 ± 6.3	31.2 ± 6.3	21.6 ± 2.3	23.7 ± 4.0	1.70 ± 0.13	1.81 ± 0.16	61.6 ± 8.6	69.7 ± 12.3	15.5	7.6 ± 5.8
Major 2015^5^	52	25	100%	24.6 ± 5.1	26.5 ± 5.4	22.9 ± 2.4	23.7 ± 3.3	1.95 ± 0.14	1.96 ± 0.18	54.8 ± 9.3	68.4 ± 10.9	18.9 ± 6.7	> 6
Mirea 2016^50^°	30	22	100%	17.8 ± 4	20.1 ± 4.6	18.8 ± 2.2	21.8 ± 2.2	1.61 ± 0.2	1.77 ± 0.2	64 ± 12	69 ± 15	13.6	5.3 ± 3.8
Moro 2013^51^	21	36	100%	22	30	24.3 ± 0.41	23.8 ± 0.33	1.92 ± 0.02	1.95 ± 0.03	56.5 ± 4.92	68.4 ± 9.31	26	NA
Moro 2013^51^ ◄	19		100%	43		21.9 ± 0.45		1.78 ± 0.02		55.3 ± 9.18		15	NA
Moro 2013^51^ ◄	17		100%	26		22.3 ± 0.43		1.81 ± 0.03		55.5 ± 6.50		24	NA
Pagourelias 2013^52^	80	26	100%	31.3 ± 10.4	26.6 ± 5.6	21.9 ± 2.5	21.2 ± 2.8	1.98 ± 0.15	2.01 ± 0.16	50 ± 7	65 ± 9	14.6 ± 5.4	11.3 ± 8.3
Pelà 2004^10^	20	15	100%	26 ± 5	26 ± 2	NA	NA	1.98 ± 0.10	1.90 ± 0.10	55 ± 11	69 ± 9	ELITE	NA
Popple 2018^53^	100	20	100%	25 ± 5	25 ± 4	NA	NA	2.0 ± 0.1	2.0 ± 0.1	NA	NA	> 20	NA
Popple 2018^53^°	100	19	100%	16 ± 1	16 ± 1	NA	NA	1.9 ± 0.1	1.8 ± 0.1	NA	NA	> 15	NA
Rimensberger 2014^54^	26	33	100%	44 ± 9	41 ± 7	22.8 ± 1.6	23.6 ± 2.1	1.9 ± 0.2	1.9 ± 0.1	56 ± 8	60 ± 7	> 10	> 5
Rundqvist 2017^55^°	27	27	ND	15.5 (13–19)	15.4 (13–19)	NA	NA	1.66 (1.37–1.98)	1.72(1.26–2.23)	63 (42–85)	71 (49–88)	> 2.5	> 2
Sanchis-Gormar 2016^56^	42	18	100%	55 ± 9	58 ± 5	NA	NA	NA	NA	54 ± 9	63 ± 8	10.6 ± 4.2	24 ± 9
Sanchis-Gormar 2016^56^ ◄	11		100%	54 ± 4		NA	NA	NA	NA	58 ± 8		10.6 ± 3.1	24 ± 9
Sanz-Delagarza2017^57^	20	20	100%	38 ± 3.5	36.2 ± 3.5	NA	NA	1.88 ± 0.09	1.91 ± 0.17	57.5 ± 8.1	66.5 ± 8.9	13.5 ± 1.6	10.2 ± 3.4
Sanz-Delagarza2017^57^ ♀	20	20	0%	37.4 ± 6.3	36.9 ± 4.6	NA	NA	1.60 ± 0.10	1.62 ± 0.11	59.9 ± 5.1	74.9 ± 7.6	13.2 ± 1.4	10.8 ± 4.0
Simsek 2013^58^	44	30	73%	24.10 ± 2.90	23.80 ± 2.10	22.6 ± 2.2	23.1 ± 2.4	NA	NA	55.22 ± 5.20	66.40 ± 5.80	14 – 18	NA
Sitges 2017^59^	100	50	100%	25 ± 6	25 ± 4	NA	NA	2.02 ± 0.21	1.92 ± 0.12	58 ± 10	65 ± 10	18	> 5
Teske 2009^4^	59	62	51%	27 ± 5.4	27.9 ± 5.6	NA	NA	1.90 ± 0.15	1.88 ± 0.25	50.7 ± 7.3	59.3 ± 10.6	12.4 ± 2.3	> 5
Teske 2009^4^ ◄	62		71%	27 ± 4.7		NA	NA	1.96 ± 0.17		50.4 ± 9.0		24.4 ± 5.4	> 5
Teske 2009^4^ ◄	54	32	72%	50 ± 6.5	45.5 ± 4.3	NA	NA	1.96 ± 0.17	1.89 ± 0.20	50.1 ± 11.1	59.8 ± 8.6	11.9 ± 3.8	> 12
Utomi 2015^60^	19	21	100%	34 ± 5	27 ± 8	NA	NA	2.1 ± 0.2	2.1 ± 0.2	56 ± 11	63 ± 10	> 12	> 11
Vitarelli 2013^61^	35	35	100%	28.7 ± 10.7	28.3 ± 11.4	22.7 ± 6	22.4 ± 2	1.91 ± 0.15	1.89 ± 0.23	54.1 ± 5.6	69.5 ± 9.1	> 15	9.6 ± 2.9
Results	2102	1311	76%	26.6 ± 8.6	26.6 ± 7.6	24.8 ± 2.01	22.89 ± 1.32	1.86 ± 0.20	1.82 ± 0.15	57.3 ± 7.06	69.6 ± 5.26		

♀Data on female extracted from article with mixed population (including both sex).

°Data on athletes aged < 18 extracted from article with age mixed population.

◄Different athletes population analysed in the same article with the same sedentary control.

NA, not available; M%, percentage of male.

h/w = hours/week.

**Table 2:** Population demographics in CMR studies.

Author name	N Athletes	N Sedentary	M% Athletes	Age Athletes	Age Sedentary	BMI Athletes (h/m^2^)	BMI Sedentary (h/m^2^)	BSA Athletes (m^2^)	BSA Sedentary (m^2^)	HR Athletes	HR Sedentary	Intensity of training (h/w)	Years of training
Arviddson 2017^7^	14	25	43%	24 (18–30)	28 (23–63)	NA	NA	1.7 (1.46–2.10)	1.82 (1.45–2.29)	50 (38–69)	62 (52–65)	ELITE	ND
Barczuk-Falecka 2018^32^°	36	24	100%	10 ± 1.4	10.4 ± 1.7	NA	NA	1.18 ± 0.21	1.23 ± 0.22	ND	ND	2 × 90minxweek + 1 × 60 min	> 2
Bernardino 2020^6^	89	77	47%	34 ± 6.1	33 ± 3.8	NA	NA	1.78 ± 0.19	1.86 ± 0.20	65.5 ± 10.2	54.4 ± 11.9	> 10	> 5
Bohm 2016^8^	33	33	100%	47 ± 8	46 ± 9	NA	NA	1.96 ± 0.1	2.00 ± 0.1	48 ± 7	65 ± 11	16.7 ± 4.4	23 ± 8
Brosnan 2015^62^	68	41	ND	35.0 ± 8.1	36.9 ± 12.6	NA	NA	1.9 ± 0.1	1.9 ± 0.2	55 ± 9	69 ± 15	> 10	10 ± 9
Domenech-Ximenos 2020^63^	49	42	100%	37 ± 5.5	34.5 ± 3.4	NA	NA	1.91 ± 0.13	1.98 ± 0.14	63.6 ± 10.3	77.3 ± 11.4	> 12	13.33 ± 7.6
Domonech-Ximenos 2020^63^ ♀	44	30	0%	34 ± 5.8	33 ± 3.7	NA	NA	1.63 ± 0.09	1.67 ± 0.12	60.15 ± 10.4	81.8 ± 13.4	> 12	14.2 ± 7.9
Dupont 2017^64^	12	12	100%	32.3 ± 7.1	33.1 ± 8.8	22.60 ± 1.06	24.9 ± 3.98	1.93 ± 0.11	2.00 ± 0.18	55 ± 11	73 ± 11	9.67 ± 2.43	ND
Esch 2010^65^	8	8	100%	31.0 ± 9.7	33.0 ± 7.3	NA	NA	NA	NA	57.3 ± 6.2	68.4 ± 15.3	11.5 ± 3.1	11.1 ± 4.5
La Gerche 2011^21^	39	14	90%	36 ± 8	38 ± 6	NA	NA	NA	NA	59 ± 9	71 ± 10	16.3 ± 5.1	10 ± 9
La Gerche 2015^16^	10	7	100%	35 ± 6	34 ± 16	22.9 ± 1.5	23.2 ± 1.7	NA	NA	52 ± 8	62 ± 12	11 (6–15)	9 (6–17)
Luijkx 2012^66^	93	56	100%	24 ± 4.3	27 ± 5.1	NA	ND	1.95 ± 0.15	2.00 ± 0.14	NA	NA	16 ± 6.1	NA
Luijkx 2012^66^ ♀	51	58	0%	23 ± 5.9	26 ± 5.8	NA	NA	1.74 ± 0.11	1.74 ± 0.11	NA	NA	13 ± 5.1	NA
Luijkx 2012^66^ ♀ ◄	24		0%	27 ± 4.4		NA	NA	1.83 ± 0.13		NA	NA	21 ± 4.3	NA
Luijkx 2012^66^ ◄	57		100%	27 ± 5		NA	NA	2.02 ± 0.16		NA	NA	23 ± 5.9	NA
Lukasz A Malek 2019^67^	30	10	100%	40.9 ± 6.6	40.0 ± 8.2	22.7 ± 1.5	26.1 ± 1.5	1.89 ± 0.07	2.00 ± 0.11	54.9 ± 9.2	69.6 ± 11.0	ELITE	6 (5–8)
Perseghin 2007^9^	9	10	100%	26 ± 5	25 ± 2	21.1 ± 1.2	22.4 ± 1.7	1.83 ± 0.13	1,93 ± 0.12	46 ± 11	64 ± 15	ELITE	NA
Prakken 2010^68^		3	100%	26 ± 6.4	26 ± 5.2	NA	NA	2.0 ± 0.2	2.0 ± 0.1	59 ± 9.7	66 ± 12	12 ± 2.4	5.1 ± 4.7
Prakken 2010^68^◄	46		100%	26 ± 4.9		NA	NA	2.0 ± 0.2		58 ± 11		24 ± 5.6	7.5 ± 4.0
Prakken 2010^68^ ♀	60	58	0%	25 ± 5.3	26 ± 5.9	NA	NA	1.8 ± 0.1	1.7 ± 0.1	58 ± 9.9	67 ± 9.8	13 ± 2.4	4.4
Prakken 2010^68^ ♀ ◄	33		0%	25 ± 4.2		NA	NA	1.9 ± 0.1		57 ± 8.6		22 ± 3.5	8.8 ± 3.7
Prakken 2011^69^	55	32	100%	50 ± 6.6	47 ± 6.6	NA	NA	1.9 ± 0.1	2.1 ± 0.2	57 ± 10	62 ± 8.9	13 ± 2.9	> 1
Prakken 2011^69^ ♀	23	33	0%	50 ± 6.5	48 ± 5.2	NA	NA	1.7 ± 0.1	1.8 ± 0.1			10 ± 1.8	> 1
Sanchis-Gormar 2016^56^	42	18	100%	55 ± 9	58 ± 5	NA	NA	NA	NA	54 ± 9	63 ± 8	10.6 ± 4.2	24 ± 9
Sanchis-Gormar 2016^56^ ◄	11		100%	54 ± 4		NA	NA	NA	NA	58 ± 8		10.6 ± 3.1	29 ± 9
Scharf 2010^33^	29	29	100%	24.6 ± 3.9	27.0 ± 3.7	NA	NA	2.03 ± 0.8	1.99 ± 0.2	56.0 ± 8.5	69.3 ± 10.3	> 3	> 10
Scharhag 2002^70^	21	21	100%	27 ± 5	26 ± 3	NA	NA	1,87 ± 0.14	1.88 ± 0.14	53 ± 6	66 ± 8	ELITE	NA
Schiros 2013^71^	19	24	53%	39 ± 10	45 ± 8	NA	NA	1.78 ± 0.23	1.91 ± 0.17	55 ± 9	67 ± 11	ELITE	> 2
Steding-Ehrenborg 2015^72^	8	7	100%	64 ± 4	66 ± 4	NA	NA	1.91 ± 0.15	1.98 ± 0.11	NA	NA	> 5	30
Swoboda 2016^73^	35	35	77%	31.3 ± 7.6	30.6 ± 8.5	22.3 ± 1.9	24.5 ± 3.3	NA	NA	NA	NA	11.5 ± 3.7	8.4 ± 6.0
Szelid 2015^74^	94	109	100%	26.9 ± 5.7	27.1 ± 5.2	25.8 ± 2.2	24.1 ± 4.6	2.38 ± 0.1	2.08 ± 0.3	NA	NA	NA	> 10
Szelid 2015^74^ ♀	32	46	0%	24.9 ± 5.3	28.0 ± 5.7	23.4 ± 2.5	21.6 ± 3.9	1.89 ± 0.3	1.74 ± 0.2	NA	NA	NA	> 10
Results	1257	909	72%	31.5 ± 9.8	31.9 ± 8.8	22.4 ± 0.5	24.4 ± 1.1	1.89 ± 0.19	1.87 ± 0.15	57.7 ± 4.35	63.4 ± 6.9		

♀Data on female extracted from article with mixed population (including both sex).

°Data on athletes aged < 18 extracted from article with age mixed population.

◄Different athletes population analysed in the same article with the same sedentary control.

NA, not available; M%, percentage of male; h/w, hours/week.

### Outcome of interest

Age showed evidence of statistically significant moderate negative association to RV E/A in athletes (*P* = 0.009, *r* = − 0.63). Age showed also a statistically significant negative and strong association to RVED longitudinal in athletes (*P* = 0.01, *r* = − 0.7). Age showed evidence of statistically significant strong positive association with an increased rate of progression to RAA both in the athletes and sedentary controls (*P* = 0.04, *r* = 0.77, *P* = 0.02, *r* = 0.85, respectively). BSA showed statistically significant strong positive association with an increased rate of progression to RVED middle in athletes (*P* = 0.001, *r* = 0.71), while in sedentary subject there was evidence of statistically significant moderate positive association with an increased rate of progression (*P* = 0.05, *r* = 0.54). Also, BSA showed statistically significant strong positive association with an increased rate of progression to RAA both in athletes and sedentary controls (*P* = 0.05, *r* = 0.82, *P* = 0.04, *r* = 0.83, respectively). Notably, BSA showed statistically significant strong negative association to ratio RV E/A in athletes (*P* = 0.002, *r* = − 0.72). With regard to ECHO data, intensity of training showed evidence of statistically significant strong positive association with an increased rate of progression to RVESA (*P* = 0.03, *r* = 0.85). Considering CMR data, the intensity of training presented statistically significant positive and, respectively, strong moderate association to RVEDVI and RVESVI (*P* = 0.01, *r* = 0.65, *P* = 0.01, *r* = 0.70, respectively). Moreover, evidence of a statistically significant moderate negative association was found between the intensity of training and EF % (*P* = 0.01, *r* = − 0.60). The multivariate analysis is shown in Table 3 and supplemental Table 1 (ECHO data) and in Table 4 (CMR data).

**Table 3:** Multivariate analysis of ECHO data. Only statistically significant data were shown.

Dimension	AGE				BSA (m^2^)				Intensity of training (h/w)	
	Athletes		Sedentary		Athletes		Sedentary		Athletes	
	*P* value	*r*	*P* value	*r*	*P* value	*r*	*P* value	*r*	*P* value	*r*
RVED longitudinal (mm)	0.0114	− 0.6992	0.6259	− 0.5544	0.706	0.5556	0.743	− 0.112	0.886	0.0609
RVESA (cm)	0.3132	0.3184	0.2724	0.4105	0.1785	0.1309	0.1959	0.555	0.0308	0.853
RVESAI (cm^2^/m^2^)	0.3663	− 0.8389	0.3182	− 0.8777	0.0415	− 0.9979	0.0117	− 0.9998	NA	NA
RV E/A	0.0091	− 0.6284	0.106	− 0.4898	0.0016	− 0.7212	0.078	− 0.5275	0.1688	0.4059
RAA (cm^2^)	0.0438	0.768	0.0166	0.8455	0.0457	0.8199	0.0423	0.8269	0.4773	− 0.7319

NA, not available; h/w, hours/week.

**Table 4:** Multivariate analysis of CMR data.

Dimension	AGE				BSA				Intensity of training (h/w)	
	Athletes		Sedentary		Athletes		Sedentary		Athletes	
	*P* value	*r*	*P* value	*r*	*P* value	r	*P* value	*r*	*P* value	*r*
RVEDV (ml)	0.9485	0.0146	0.5797	− 0.1695	0.0569	0.5883	0.2967	0.392	0.1159	0.5017
RVEDVI (ml/m^2^)	0.726	− 0.0951	0.4515	− 0.1876	0.0887	0.3715	0.349	0.2345	0.0084	0.6524
RVESV (ml)	0.5942	− 0.1096	0.3029	− 0.4179	0.1133	0.7109	0.596	0.404	0.0751	0.5859
RVESVI (ml/m^2^)	0.1387	− 0.4762	0.1221	− 0.3896	0.1318	0.3584	0.3421	0.2638	0.0072	0.7043
RVSV (ml)	0.0671	− 0.3973	0.8966	0.069	0.8888	− 0.1112	0.8364	0.1636	0.874	0.1966
RVSVI (ml/m^2^)	0.9695	− 0.0203	0.0674	− 0.5987	0.4555	0.2861	0.7691	0.1146	NA	NA
EF %	0.6754	− 0.1518	0.1485	0.3111	0.7527	− 0.0712	0.5675	− 0.1444	0.0103	− 0.6035
Mass	0.0524	0.3703	0.9772	− 0.0152	0.2925	− 0.4655	0.7043	0.2344	0.9712	− 0.0226
Mass index	0.2581	0.4543	0.2318	− 0.3417	0.1315	0.3809	0.2845	0.3212	0.3042	0.3414

NA, not available; h/w, hours/week.

A statistically significant difference was observed between RVEDVI means difference values in male and RVEDVI means difference values in female (*P* = 0.001), as shown in Table 5.

**Table 5:** Comparison between increment of CMR values among sex (only studies with both male and female data were shown separately).

Dimension	*P* value
RVEDVI	0.001
RVESVI	0.175
EF %	0.423
MASS index	0.28

A statistically significant difference was found between all RV ECHO and CMR means in endurance athletes and RV reference means. Results are shown in Table 6 and in Table 7.

**Table 6:** T test between ECHO means of athletes and reference means.

Dimension	N athletes	Mean	*P* value
RVEDbasal	895	39.71 ± 3.2	< 0.0001
RVEDmiddle	1160	32.86 ± 2.52	< 0.0001
RVEDlongitudinal	822	82 ± 7.59	< 0.0001
RVEDA male	522	29.87 ± 8.7	< 0.0001
RVESA male	230	17.37 ± 6.64	< 0.0001
RVWT	828	4.24 ± 0.99	< 0.0001
RVOT1	179	30.24 ± 1.11	< 0.0001
RVOT1/BSA	410	20.18 ± 4	
RVOT2	803	31.83 ± 2.34	< 0.0001
RVOT2/BSA	348	22.3 ± 3.15	
RVOT3	574	26.78 ± 1.46	< 0.0001
RVOT3/BSA	139	20 ± 0.53	
TAPSE	986	24.94 ± 1.93	< 0.0001
RV E/A	1032	1.9 5 ± 0.26	< 0.0001
FAC%	632	46.35 ± 3.62	< 0.0001

**Table 7:** T test between CMR means (only studies with both male and female data shown separately) and reference means.

Dimension	N athletes	Mean	*P* value
RVEDVI male	477	132.25 ± 9.63	< 0.0001
RVEDVI female	267	109.5 ± 9.35	< 0.0001
RVESVI male	477	64.21 ± 7.06	< 0.0001
RVESVI female	267	51.61 ± 5.95	< 0.0001
EF % male	477	51.89 ± 2.75	< 0.0001
EF % female	267	53.85 ± 2.68	< 0.0001
MASS index male	428	17.99 ± 7.78	< 0.0001
MASS index female	223	14.43 ± 3.77	< 0.0001

In athletes, EF % showed evidence of a statistically significant strong negative association to RVEDV and RVESV (*P* = 0.01, *r* = − 0.70, *P* = 0.0003, *r* =  − 0.89, respectively). Data are reported in Table 8.

**Table 8:** Multivariate analysis between EF % and ventricular chamber volumes in athletes.

Dimension	*P* value	*r*
RVEDV (ml)	0.0072	− 0.7043
RVESV (ml)	0.0003	− 0.888
RVSV (ml)	0.0837	− 0.8275

## Discussion

Endurance athletes demonstrated a statistically significant association between RV remodelling and age, BSA and intensity of training. 2-D ECHO is the easiest way to assess morphological and functional parameters of the right heart, and CMR became fundamental to evaluate the RV morphology^23^. In fact, it is currently used to diagnose ARVC, although normal range values of both ECHO and CMR parameters in endurance athletes have not been established yet^30^. Right cardiac chambers remodelling depends on many factors. To analyse the differences between male and female RV adaptation to exercise training, five CMR studies in which data were separated for sex were selected. Changes in morphological and functional parameter both in males (athletes versus sedentary) and in females (athletes versus sedentary) were analysed. There are no differences in the kinetics of trend of adaptation between males and females, with the exception of RVEDV, and all the other parameters showed a similar trend of adaptation both in female and male endurance athletes. Age and increased BSA in athletes are linked to a decrease in diastolic function with a reduction in RV E/A. The intensity of exercise training is associated with heart morphological remodelling. The studies show that 6–8 h/w are sufficient to induce remodelling evident on ECHO and CMR^31–33^. Another goal of this systematic review was to discuss whether the normal RV reference values fitted the RV characteristics in endurance athletes. Athletes involved in endurance sport have different ECHO parameters from those of the general population^20^. Comparing the studies’ results with the reference values, the mean values of all considered parameters in endurance athletes are higher than those of the general population indicated by the American Society of Echocardiography and the European Association of Cardiovascular Imaging^34^. Furthermore, there is a difference between the reference mean values adopted for CMR parameters and the mean values in endurance athletes^35^. This is in accordance with an earlier systematic review, which underlines how endurance sport induces the most significant modification in the right heart, with eccentric hypertrophy^12^. Such structural changes could lead to incorrectly diagnose ARVC. The ARVC is a group of cardiomyopathies with a similar phenotype and tendency to ventricular arrhythmias. Mutations encoding desmosomal genes are associated to ARVC. In some individuals the phenotypical expression can be triggered by endurance exercise training^15^. The mean values of RVEDVI are 132.25 ± 9.63 mL/m^2^ and 109.5 ± 9.35 mL/m^2^ respectively for males and females, and they are both over the limit of the major criteria for diagnosis of ARVC (≥ 110 mL/m^2^ for males and ≥ 100 mL/m^2^ for females)^30^. The value of 30.24 ± 1.11 mm for RVOT1 is below the limit of major criteria (≥ 32 mm) but it is included in the minor criteria limit (≥ 29 to ≤ 32 mm), while the BSA indexed value (20.18 ± 4 mm/m^2^) is just above the major criteria limit (≥ 19 mm/m^2^). The value of 31.83 ± 2.34 mm for RVOT2 is above the limit of the minor criteria (> 32 mm), and the RVOT2/BSA (22.3 ± 3.15 mm/m^2^) is beyond the limit of the major criteria (> 21 mm/m^2^). Functional parameters (FAC % and EF %) are not over the limit for diagnosis of ARVC (> 33% to ≤ 40% and > 40% to ≤ 45%, respectively)^28^. A low value of RV E/A is a sign of diastolic disfunction^36^. As highlighted in previous studies, age affects diastolic function, with proliferation of extracellular matrix and crosslinking of collagen fibres^4^. The present study shows that a negative association is evident among athletes, but not among sedentary subjects. Some chronic cardiac microinjuries can emphasise the physiological mechanisms of extracellular matrix rearrangement and fibrotic alteration that normally occur with aging. This leads to a decrease in right heart wall compliance with a reduction in diastolic function. Low values in EF % are related to ventricular chamber dilatation, as shown in Table 8, with a negative association with EDV and ESV. EF % exhibits a negative association with the intensity of training. The more an athlete trains, the more the EF % decreases. Actually, in the case of ventricle dilatation, a lower EF % is necessary to obtain the same SV^22^.Although EF% decreases, this is not sufficient to justify a systolic disfunction or any other pathological condition, and it should be regarded as a physiological adaptation to the increase in EDV and ESV. To make a correct diagnosis of ARVC, it is important to examine the family history, and to fulfil the other Task Force criteria^30^. Pathology should be suspected when ECHO shows akinesia, dyskinesia, or an aneurysm, because they are not features of athlete’s heart^37^. Indeed, the remodelling induced by endurance sport could resemble a cardiomyopathy, but the real impact on future morbidity is still unknown. However, if a subject has a latent ARVC, sport-induced adaptation could deteriorate this condition^15,16^.

Cardiac remodelling is evident in both sexes, but the lack of studies investigating separately ECHO parameters in male and female athletes does not allow to thoroughly assess similarity and differences. Also, atrial adaptations should be considered, but there are still few studies which analyse atrial chamber remodelling in athletes. A positive association was shown between RAA and age, and between RAA and BSA, but the data available on RA were fewer than data on RV: other studies should be conducted to assess the impact of RA remodelling in endurance athletes. Similarly, years of training were not taken into account because of the lack of relevant data, and further investigations could be conducted to ascertain how many years of training induce permanent RV modifications. The impact of systolic and diastolic disfunction should be analysed considering a longer follow-up period to estimate the incidence of cardiovascular diseases in endurance athletes. Future studies should overcome these limitations to offer a more complete overview.

## Conclusion

Endurance athletes exhibit an association between RV remodelling and age, BSA and intensity of training. In particular, RV E/A is an age-related functional parameter, and it should be carefully evaluated because it is a marker of diastolic dysfunction. The systolic function of the RV is decreased in endurance athletes. This is a normal adaptation to exercise, induced by structural changes, and it is within the normal limits. RV ECHO and CMR values in endurance athletes are slightly different from the reference values established in the general population. This could lead to an incorrect diagnosis of ARVC.

## Supplementary Information

Supplementary Information 1.

Supplementary Information 2.
