# Supplementary material for: Right heart exercise-training-adaptation and remodelling in endurance athletes
Source: Sci Rep. 2021 Nov 18;11:22532. doi: 10.1038/s41598-021-02028-1 (PMC8602371; doi:10.1038/s41598-021-02028-1)
Supplement: Supplementary file 2 — Supplementary Information 2. [file 41598_2021_2028_MOESM2_ESM.docx]

| Dimension | AGE | | | | BSA (m²) | | | | Intensity of training (h/w) | |
| --- | --- | --- | --- | --- | --- | --- | --- | --- | --- | --- |
|  | **Athletes** | | **Sedentary** | | **Athletes** | | **Sedentary** | | **Athletes** | |
|  | **P value** | ***r*** | **P value** | ***r*** | **P value** | ***r*** | **P value** | ***r*** | **P value** | ***r*** |
| RVED basal (mm) | 0.7931 | 0.08 | 0.7938 | -0.0894 | 0.0925 | 0.5315 | 0.7816 | 0.1009 | 0.3912 | - 0.4329 |
| RVEDI basal (mm/m²) | 0.9186 | 0.1274 | 0.982 | 0.0283 | 0.8883 | - 0.1745 | 0.7904 | - 0.3233 | NA | NA |
| RVED middle (mm) | 0.0782 | 0.4029 | 0.7272 | 0.1628 | 0.0009 | 0.7108 | 0.047 | 0.5384 | 0.6635 | - 0.1483 |
| RVEDI middle (mm/m²) | 0.7148 | - 0.2259 | 0.2702 | -0.9113 | 0.2081 | - 0.2081 | 0.2797 | - 0.7203 | 0.2585 | - 0.7415 |
| RVED longitudinal (mm) | 0.0114 | - 0.6992 | 0.6259 | -0.5544 | 0.706 | 0.5556 | 0.743 | - 0.112 | 0.886 | 0.0609 |
| RVEDA (cm²) | 0.2992 | 0.269 | 0.4503 | 0.2297 | 0.2165 | - 0.3525 | 0.5542 | 0.2006 | 0.0619 | 0.6829 |
| RVEDAI (cm²/m²) | 0.6561 | 0.1306 | 0.9474 | 0.0204 | 0.6039 | - 0.152 | 0.4895 | 0.2108 | 0.4579 | - 0.308 |
| RVESA (cm) | 0.3132 | 0.3184 | 0.2724 | 0.4105 | 0.1785 | 0.1309 | 0.1959 | 0.555 | 0.0308 | 0.853 |
| RVESAI (cm²/m²) | 0.3663 | - 0.8389 | 0.3182 | -0.8777 | 0.0415 | - 0.9979 | 0.0117 | - 0.9998 | NA | NA |
| RVEF % | 0.5645 | 0.2228 | 0.9718 | 0.0151 | 0.0916 | - 0.6818 | 0.1204 | - 0.7014 | 0.8662 | 0.1053 |
| RV E/A | 0.0091 | - 0.6284 | 0.106 | -0.4898 | 0.0016 | - 0.7212 | 0.078 | - 0.5275 | 0.1688 | 0.4059 |
| RVWT (mm) | 0.9339 | - 0.0302 | 0.3206 | 0.3746 | 0.7686 | - 0.1148 | 0.9326 | 0.034 | 0.0734 | 0.8422 |
| TAPSE (mm) | 0.0924 | 0.4499 | 0.3339 | 0.2915 | 0.0658 | 0.5468 | 0.4525 | 0.2689 | 0.4592 | - 0.2839 |
| RAA (cm²) | 0.0438 | 0.768 | 0.0166 | 0.8455 | 0.0457 | 0.8199 | 0.0423 | 0.8269 | 0.4773 | - 0.7319 |
| RAAI (cm²/m²) | 0.5705 | - 0.4295 | 0.9123 | 0.1373 | 0.9222 | - 0.0778 | 0.7257 | - 0.4177 | 0.0881 | 0.9904 |
| RAV (ml) | NA | NA | NA | NA | NA | NA | NA | NA | NA | NA |
| RAVI (ml/m²) | 0.1344 | 0.5074 | 0.2541 | 0.3984 | 0.2877 | 0.3735 | 0.1654 | 0.475 | 0.1854 | 0.5211 |
| FAC % | 0.1708 | 0.4042 | 0.623 | 0.1507 | 0.1242 | 0.4688 | 0.5815 | - 0.1773 | 0.4123 | 0.3712 |
| RVOT1 (mm) | 0.7428 | 0.258 | 0.6489 | 0.3511 | 0.4269 | 0.5731 | 0.1902 | 0.8098 | NA | NA |
| RVOT1 BSA (mm/m²) | 0.8755 | 0.0666 | 0.2584 | - 0.4411 | 0.4981 | 0.2823 | 0.8758 | 0.0733 | 0.3774 | - 0.5124 |
| RVOT2 (mm) | 0.5243 | 0.2042 | 0.2404 | 0.4695 | 0.5799 | - 0.188 | 0.0099 | 0.875 | 0.5538 | - 0.6449 |
| RVOT2 BSA (mm/m²) | 0.7168 | - 0.1692 | 0.8449 | - 0.0918 | 0.2229 | 0.5282 | 0.7061 | 0.1758 | NA | NA |
| RVOT3 (mm) | 0.3214 | 0.5646 | 0.2589 | 0.6257 | 0.6636 | - 0.2675 | 0.2819 | 0.6028 | NA | NA |
| RVOT3 BSA (mm/m²) | 0.7074 | - 0.4435 | 0.6143 | - 0.5695 | 0.5935 | - 0.569 | 0.2123 | - 0.9449 | NA | NA |

**Table 1 supplemental**. Multivariate analysis of ECHO data.

NA, not available; h/w, hours/week.
